# Ancestry-associated co-alteration landscape of *KRAS* and *EGFR*-altered non-squamous NSCLC

**DOI:** 10.1038/s41698-024-00644-4

**Published:** 2024-07-20

**Authors:** Saumya D. Sisoudiya, Armande Ang Houle, Tharu Fernando, Timothy R. Wilson, Jennifer L. Schutzman, Jessica Lee, Alexa Schrock, Ethan S. Sokol, Smruthy Sivakumar, Zhen Shi, Gaurav Pathria

**Affiliations:** 1 Foundation Medicine Inc., Boston, MA USA; 2 Genentech Inc., South San Francisco, CA USA; 3 Present Address: TOLREMO Therapeutics, Basel, Switzerland

**Keywords:** Predictive markers, Non-small-cell lung cancer

## Abstract

Racial/ethnic disparities mar NSCLC care and treatment outcomes. While socioeconomic factors and access to healthcare are important drivers of NSCLC disparities, a deeper understanding of genetic ancestry-associated genomic landscapes can better inform the biology and the treatment actionability for these tumors. We present a comprehensive ancestry-based prevalence and co-alteration landscape of genomic alterations and immunotherapy-associated biomarkers in patients with *KRAS* and *EGFR*-altered non-squamous (non-Sq) NSCLC. *KRAS* was the most frequently altered oncogene in European (EUR) and African (AFR), while *EGFR* alterations predominated in East Asian (EAS), South Asian (SAS), and Admixed American (AMR) groups, consistent with prior studies. As expected, *STK11* and *KEAP1* alterations co-occurred with *KRAS* alterations while showing mutual exclusivity with *EGFR* alterations. EAS and AMR *KRAS*-altered non-Sq NSCLC showed lower rates of co-occurring *STK11* and *KEAP1* alterations relative to other ancestry groups. Ancestry-specific co-alterations included the co-occurrence of *KRAS* and *GNAS* alterations in AMR, *KRAS,* and *ARID1A* alterations in SAS, and the mutual exclusivity of *KRAS* and *NF1* alterations in the EUR and AFR ancestries. Contrastingly, *EGFR*-altered tumors exhibited a more conserved co-alteration landscape across ancestries. AFR exhibited the highest tumor mutational burden, with potential therapeutic implications for these tumors.

## Introduction

Lung cancer is the second most commonly diagnosed cancer globally and is the leading cause of cancer-related deaths^1^. Non-Small Cell Lung Cancer (NSCLC) accounts for 80–85% of all lung cancers, with non-squamous (non-Sq) NSCLC, particularly lung adenocarcinoma, as the most common histologic subtype^2^. Despite declines in NSCLC-related mortality in the past decade in the US, racial and ethnic disparities persist, including minority patient groups presenting with cancer at a younger age and more advanced stage compared to White individuals^3,4^.

The advent of precision medicine and comprehensive genomic profiling using targeted sequencing has led to unique opportunities to identify the optimal treatment options for patients, requiring a growing emphasis on biomarker-driven clinical trials and a need to better define patient cohorts for these trials. As such, current drug development and patient enrollment in clinical trials are heavily influenced by our understanding of the prevalence of genomic alterations. However, due to the limited access to certain medical centers by minority groups and other historical biases, the databases utilized to inform the size of biomarker-selected patient populations are overwhelmingly composed of data from patients of Western European descent^5^. Further, minority populations including Black, Asian, Hispanic/Latino, and Indigenous Peoples represent a small fraction of the patient population characterized in the widely utilized dataset, The Cancer Genome Atlas (TCGA) (12%, 3%, 3%, <0.5% respectively), and these populations continue to be underrepresented in clinical trials^6–8^.

Genomic profiles, gene expression changes, and prognostic significance of specific biomarkers have been shown to vary by race and ancestry across different tumor types, including NSCLC^9–15^. However, the interpretation of clinical trial outcomes and real-world implications, including the identification of predictive and prognostic biomarkers, is heavily constrained in underrepresented populations^6,7,16,17^. Due to a limited understanding of biomarker prevalence in patients from minority populations, forecasting clinical trial enrollment metrics to facilitate inclusive and equitable healthcare has also been hampered. Furthermore, because of their underrepresentation in clinical studies, fuller comprehension of the implications of the clinical data, including therapeutic indexes, pharmacokinetics and pharmacodynamics, and drug safety and toxicity attributes of experimental treatments in the minority patient population groups has been challenging.

There are many contributing factors to the disparities observed in cancer outcomes and representation in clinical studies, including a general lack of trust in the medical establishment, limited awareness of cancer screening and clinical trial opportunities, long-standing effects of structural racism, and environmental factors across different racial/ethnic groups. However, the magnitude of observed differences cannot solely be attributed to socioeconomic factors^18–20^. Differences in the prevalence and the landscape of molecular alterations in different ancestry groups may also impact cancer outcomes and impact equitable representation in clinical trials^18,19^. However, the continued lack of diversity in clinical studies has led to a rather poor understanding of the contribution of genomics to the disparities in the prevalence and outcomes of non-Sq NSCLC in different ancestry groups^21,22^.

While self-reported race has been utilized to study the genetic features associated with cancer incidence and outcomes, this is often a challenge in clinical sequencing assays, where self-reported race and ethnicity information is not always available. However, using measures of genetic ancestry that can be derived from the data and provide accurate decipherment of ancestry for each patient, can play a critical role in understanding the genetic basis of cancer disparities in different populations and provide insights on targetable therapies and precision medicine efforts in diverse populations^16,23,24^. Here we sought to investigate the prevalence and co-alteration landscape of genomic alterations in non-Sq NSCLC using a diverse real-world cohort comprising patients of European (EUR), African (AFR), East Asian (EAS), South Asian (SAS), and Admixed American (AMR) ancestries, focusing our analyses on tumors with alterations in *KRAS* and *EGFR*, the two major oncogenic drivers of non-Sq NSCLC. Additionally, we present ancestry-associated patterns of programmed death-ligand 1 (PD-L1) expression and tumor mutational burden (TMB), known predictive biomarkers of response to immune checkpoint inhibitors, across the five ancestry groups to gain molecular insights into ancestry-based genomic landscapes as well as to better inform strategies for patient treatment and clinical trial enrollment.

## Results

### *KRAS* and *EGFR* alterations show varying prevalence in different ancestry groups

**Fig. 1:**
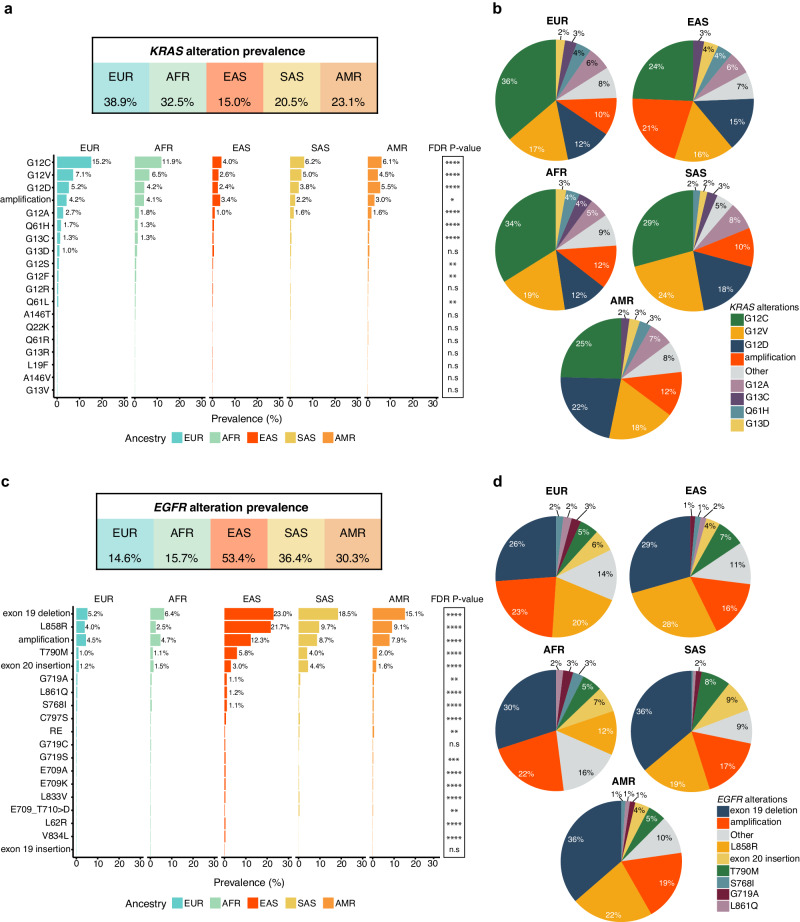
Spectrum of *KRAS* and *EGFR* alterations in non-Sq NSCLC based on ancestry. **a** The overall *KRAS* alteration prevalence (top) and the prevalence of individual *KRAS* alterations (bottom) in each ancestry group. Alterations observed in at least 50 cases in the overall non-Sq NSCLC cohort are shown. Statistically significant patterns, determined by a Chi-squared test followed by FDR correction are shown with the following *P-*value thresholds: * 0.05, ** 0.01, *** 0.001, **** 0.0001, n.s not significant. **b** Pie chart displaying the breakdown of the different *KRAS* alterations observed in each ancestry group. The color and percentages denote the fraction of specific *KRAS* alterations relative to all the *KRAS* alterations detected. **c** The overall *EGFR* alteration prevalence (top) and the prevalence of individual *EGFR* alterations (bottom) in each ancestry group. Alterations observed in at least 50 cases across all ancestry groups are shown. Statistically significant patterns, determined by a Chi-squared test followed by FDR correction, are shown with the following *P-*value thresholds: * 0.05, ** 0.01, *** 0.001, **** 0.0001, n.s not significant. **d** Pie chart displaying the breakdown of the different *EGFR* alterations observed in each ancestry group. The color and percentages denote the fraction of specific *EGFR* alterations relative to all the *KRAS* alterations detected. (EUR: European, AFR: African, EAS: East Asian, SAS: South Asian, AMR: Admixed American).

A total of 68,297 adult patients with non-Sq NSCLC who received tissue biopsy-based comprehensive genomic profiling (CGP) using FoundationOne® (n = 24,042) or FoundationOne®CDx (n = 44,255) during routine clinical care were included in this study. This US-based cohort included 55,430 patients of EUR ancestry (81%), 7062 patients of AFR ancestry (10%), 3297 patients of EAS ancestry (5%), 2011 patients of AMR ancestry (3%), and 497 patients of SAS ancestry (<1%). The overall genomic landscape of non-Sq NSCLC varied by ancestry (Supplementary Fig. 1, Supplementary Data 1). As expected, *KRAS* and *EGFR* were the most frequently altered oncogenes in the overall dataset^25^, with additional ancestry-associated patterns. In EUR and AFR ancestry, *KRAS* was the most frequently altered oncogene (38.9% EUR, 32.5% AFR) followed by *EGFR* (14.6% EUR, 15.7% AFR) (Supplementary Fig. 1, Supplementary Data 1). In contrast, for EAS, SAS as well as AMR subgroups, *EGFR* was the most frequently altered oncogene (53.4% EAS, 36.4% SAS, 30.3% AMR), with *KRAS* alterations being relatively less prevalent (15.0% EAS, 20.5% SAS, 23.1% AMR) (Supplementary Fig. 1, Supplementary Data 1). *KRAS* G12C was the most prevalent *KRAS* alteration across all ancestry groups, with a higher prevalence in EUR (15.2%) and AFR (11.9%) groups compared to other ancestry groups (4–6%) (Fig. 1a, Supplementary Data 2). In comparison, *KRAS* G12D and G12V alterations, while rare in EAS (2.4%), were observed at similar rates (~4–6%) across other ancestry groups (Fig. 1a, Supplementary Data 2). Notably, the significantly lower rate of *KRAS* G12C in EAS, SAS, and AMR ancestry was the predominant contributor to the lower observed prevalence of overall *KRAS* alterations in these ancestry groups compared to EUR and AFR ancestry groups (Fig. 1b). In comparison to base substitutions, *KRAS* amplifications were less common (4.1% prevalence across ancestry groups). Interestingly *KRAS* amplifications represent 21% of all *KRAS* alterations in the EAS ancestry group, which is higher than the rates observed in the other ancestry groups (Fig. 1a, b, Supplementary Data 2). Of note, samples with *KRAS* amplifications frequently harbored concurrent *KRAS* short variants across all ancestries; 65.1% EUR, 55.6% AFR, 48.3% AMR, 40.2% EAS and 36.4% SAS *KRAS*-amplified cases also had a *KRAS* short variant (Supplementary Fig. 2A).

In contrast to *KRAS*, *EGFR* alterations were most commonly seen in patients of EAS ancestry (53.4%), with exon 19 deletions and L858R being the most prevalent *EGFR* alterations across all ancestry groups (Fig. 1c, Supplementary Data 3). While these two alteration groups occurred at similar rates in EAS and EUR populations (~23–29% of all *EGFR* alterations), L858R alterations were less common than exon 19 deletions in SAS, AMR, and AFR populations (~19–22% vs. 30–38% of all *EGFR* alterations respectively; Fig. 1d, Supplementary Data 3). *EGFR* amplifications were also commonly observed across ancestry groups: 12.3% EAS, 8.7% SAS, 7.9% AMR, 4.7% AFR, and 4.5% EUR (Fig. 1c, Supplementary Data 3). While EAS, SAS and AMR populations displayed a high prevalence of *EGFR* amplifications, >90% of EAS and SAS cases and >80% of AMR cases with *EGFR* amplifications had concomitant *EGFR* short variants (Supplementary Fig. 2B). In comparison, only 50–60% of EUR and AFR non-Sq NSCLC with *EGFR* amplification showed concomitant *EGFR* short variants (Supplementary Fig. 2B). In addition to these alterations, exon 20 insertions, albeit overall rare, were more common in SAS population (4.4% of samples, ~9% of all *EGFR* alterations) compared to other ancestry groups (Fig. 1c, d, Supplementary Data 3).

### Co-alteration landscape of *KRAS* and *EGFR*-altered non-Sq NSCLC reveals unique ancestry-specific patterns

**Fig. 2:**
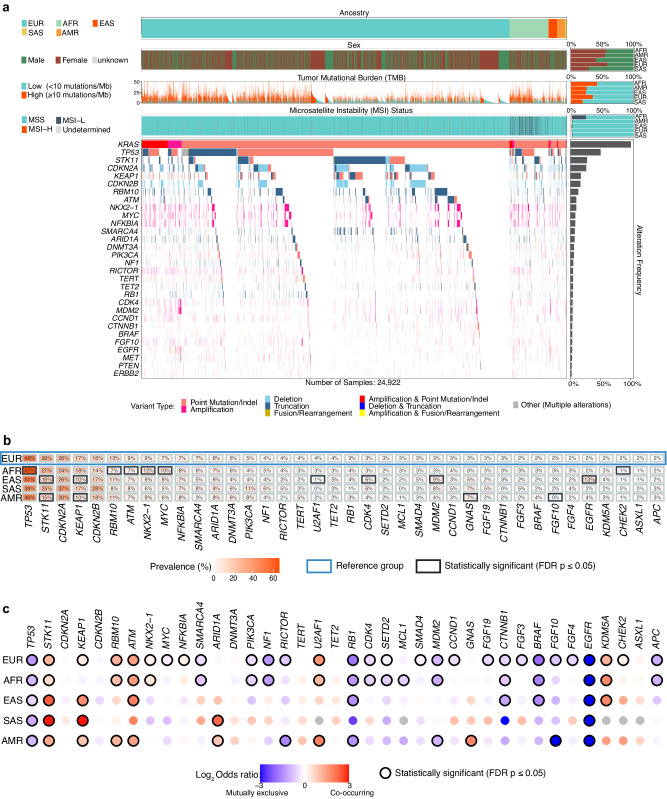
Co-alteration landscape of *KRAS*-altered non-Sq NSCLC based on ancestry. **a** Tileplot showing the overall mutational spectrum of *KRAS*-altered non-Sq NSCLC with alterations in the top 30 most frequently occurring genes displayed. Patterns of tumor mutational burden (TMB), microsatellite instability (MSI), and sample-level annotations of ancestry and sex are annotated. The gene alterations within a sample are colored based on the type of alteration. **b** Comparative prevalence of co-occurring gene alterations in *KRAS*-altered non-Sq NSCLC based on ancestry. Genes with a prevalence of at least 2% across all *KRAS-*altered cases are shown. The prevalence of a gene alteration in each ancestry was compared against European ancestry. Statistically significant patterns, determined by a Fisher’s exact test with FDR correction (FDR p ≤ 0.05), are highlighted with a black box. **c** Patterns of co-occurrence and mutual exclusivity between *KRAS* and other gene alterations in each ancestry. Genes with a prevalence of at least 2% across all *KRAS-*altered cases are shown. Statistically significant patterns, determined by a Fisher’s exact test with FDR correction (FDR p ≤ 0.05), are highlighted with a black circle. Patterns of co-occurrence are shown in shades of red while patterns of mutual exclusivity are shown in shades of blue. (EUR: European, AFR: African, EAS: East Asian, SAS: South Asian, AMR: Admixed American).

Among the 24,922 non-Sq NSCLC harboring *KRAS* alterations, the most common co-alterations included loss-of-function alterations in *TP53*, *STK11*, *KEAP1,* and *CDKN2A/B* (Fig. 2a, Supplementary Data 4). Interestingly, AFR showed a higher rate of *TP53* co-alteration compared to EUR (60.7% vs. 48.8%, p < 10^−5^; Fig. 2b, Supplementary Data 4). Despite *TP53* being the most common alteration in *KRAS-*altered tumors, the prevalence was lower than in *KRAS* wildtype cases (48.8% vs. 70.7% EUR, 60.7% vs. 74.3% AFR, 51.4% vs. 60.4% EAS, 39.2% vs. 57.2% SAS, 45.9% vs. 61.8% AMR, all p < 0.05) (Fig. 2c, Supplementary Data 5). Alterations in *STK11* and *KEAP1* showed strong co-occurrence with *KRAS* alterations across all ancestry groups (Fig. 2c, Supplementary Data 5). Interestingly, both *STK11* and *KEAP1* co-alterations were less common in EAS and AMR subgroups compared to EUR, AFR, and SAS (Fig. 2b, Supplementary Data 4). Consistently, EAS and AMR also showed a lower prevalence of concomitant *KRAS*, *STK11,* and *KEAP1* alterations (~1% compared to 4.2% in EUR, Supplementary Fig. 3, Supplementary Data 6). As expected, *KRAS* alterations were mutually exclusive with alterations in other clinically actionable driver genes such as *EGFR*, *ERBB2*, *RET*, *ALK*, *MET*, *ROS1,* and *BRAF* across all ancestries^26^ (Fig. 2c, Supplementary Data 5).

Of note, despite the well-known mutual exclusivity of *KRAS* and *EGFR* alterations (Fig. 2c, Supplementary Data 5), 12.8% of the *KRAS*-altered tumors in the EAS ancestry showed co-occurring *EGFR* alterations as compared to 2–4% in the other ancestry groups (Fig. 2b, Supplementary Data 4). Upon closer examination, a vast majority of the 63 EAS cases with co-occurring *EGFR* and *KRAS* alterations had an amplification of at least one of these genes (Supplementary Data 7). Most commonly, 39 cases harboring *EGFR* L858R, or exon 19 deletions exhibited *KRAS* amplifications; 15 cases harbored amplifications in both *EGFR* and *KRAS* apart from short variants in one or more of these genes (Supplementary Data 7). The presence of amplifications in at least one of these genes may be explained by the synthetic lethality of concomitant *KRAS* and *EGFR*-activating mutations^27^. Additionally, the presence of concomitant *EGFR* and *KRAS* alterations may represent treatment resistance mechanisms within these tumors^28,29^.

Co-occurring alterations in DNA damage checkpoint kinase (*ATM*), lysine-specific demethylase (*KDM5A*), and cyclin D2 (*CCND2*) were also noted in *KRAS-*altered tumors across most ancestries (Fig. 2b, c, Supplementary Data 4, Supplementary Data 5**;** not evaluable in SAS due to limited number of cases). Apart from alterations that were consistently co-occurring or mutually exclusive with *KRAS* alterations across ancestry groups, several genomic alterations were also found to be uniquely enriched or depleted in specific ancestries (Fig. 2c, Supplementary Data 5). For example, strong co-occurrence with *KRAS* alterations was observed for *GNAS* alterations in AMR (6.9% in *KRAS*(+) vs 2.1% in *KRAS*(-) cases; OR = 3.5, p < 10^−^^5^), *ARID1A* in SAS (8.9% in *KRAS*(+) vs 2.0% in *KRAS*(-) cases; OR = 4.7, p = 0.02), while mutual exclusivity of *NF1* alterations was predominant in EUR (4.0% in *KRAS*(+) vs 9.7% in *KRAS*(-) cases; OR = 0.4, p < 10^−^^5^) and AFR (4.8% in *KRAS*(+) vs 10.8% in *KRAS*(-) cases; OR = 0.4, p < 10^−^^5^) ancestries (Fig. 2c, Supplementary Data 5). Of note, *MDM2* alterations showed mutual exclusivity with *KRAS* alterations in EUR, AFR, and AMR groups; interestingly, *MDM2* alterations occurred in 8% of *KRAS*-altered SAS cases (p = 0.63) and 9% EAS (p < 10^−^^5^) compared to only 3% of *KRAS*-altered cases in EUR (Fig. 2b, Supplementary Data 4).

Given our prior observation of frequently co-occurring *KRAS* amplifications and short variants, we studied the co-occurrence and mutual exclusivity profile of the samples exclusively harboring either *KRAS* short variants or *KRAS* amplifications. Samples with *KRAS* short variants showed a higher degree of mutually exclusive alterations compared to *KRAS*-amplified samples (Supplementary Fig. 4A, B, Supplementary Data 8, 9). For example, *MDM2* alterations (amplifications) were mutually exclusive with *KRAS* short variants, while co-occurring with *KRAS* amplifications. A similar trend was observed for other alterations, including *RICTOR*, *CDK4,* and *CCND1*. Due to the limited sample size of *KRAS*-amplified EAS, SAS, and AMR cases, we were generally underpowered for statistical evaluation of similar comparisons within these ancestry groups.

**Fig. 3:**
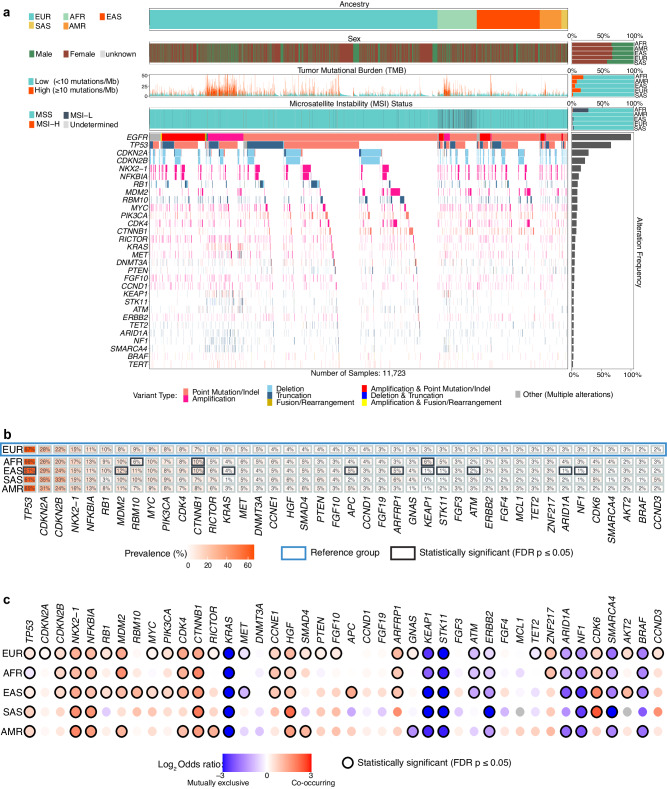
Co-alteration landscape of *EGFR*-altered non-Sq NSCLC based on ancestry. **a** Tileplot showing the overall mutational spectrum of *EGFR*-altered non-Sq NSCLC with alterations in the top 30 most frequently occurring genes displayed. Patterns of tumor mutational burden (TMB), microsatellite instability (MSI), and sample-level annotations of ancestry and sex are annotated. The gene alterations within a sample are colored based on the type of alteration. **b** Comparative prevalence of co-occurring gene alterations in *EGFR*-altered non-Sq NSCLC based on ancestry. Genes with a prevalence of at least 2% across all *EGFR-*altered cases are shown. The prevalence of a gene alteration in each ancestry was compared against European ancestry. Statistically significant patterns, determined by a Fisher’s exact test with FDR correction (FDR p ≤ 0.05), are highlighted with a black box. **c** Patterns of co-occurrence and mutual exclusivity between *EGFR* and other gene alterations in each ancestry. Genes with a prevalence of at least 2% across all *EGFR-*altered cases are shown. Statistically significant patterns, determined by a Fisher’s exact test with FDR correction (FDR p ≤ 0.05), are highlighted with a black circle. Patterns of co-occurrence are shown in shades of red while patterns of mutual exclusivity are shown in shades of blue. (EUR: European, AFR: African, EAS: East Asian, SAS: South Asian, AMR: Admixed American).

We also interrogated the co-alteration landscape of *EGFR-*altered tumors (Fig. 3a, Supplementary Data 10). As with *KRA*S-altered tumors and owing to its high overall prevalence in non-Sq NSCLC, *TP53* alterations were the most frequent in *EGFR*-mutant tumors across all ancestries (61–67%), followed by *CDKN2A* (26–31%) and *CDKN2B* alterations (24–33%) (Fig. 3a, b, Supplementary Data 10). *STK11* and *KEAP1* alterations that frequently co-occurred with *KRAS* mutations, showed strong mutual exclusivity with *EGFR* mutations across all ancestry groups (p < 10^−^^5^) (Fig. 3c, Supplementary Data 11). Despite this general mutual exclusivity, *EGFR*-altered AFR cases showed a higher rate of *KEAP1* co-alterations than EUR (6.1% vs. 3.3%, p = 0.003), while *EGFR*-altered EAS cases showed a much lower rate of *KEAP1* and *STK11* alterations than EUR (~1% vs ~3%, p < 10^−^^5^ for each) (Fig. 3b, Supplementary Data 10).

*MDM2* alterations frequently co-occurred with *EGFR* alterations across ancestries (OR > 2, p < 10^−^^5^ for all groups except SAS; Fig. 3c, Supplementary Data 11); notably, *MDM2* alterations were more frequent in *EGFR*-altered EAS as compared to the *EGFR*-altered EUR subgroup (12.3% vs 8.3%, p < 10^−^^5^) (Fig. 3b, Supplementary Data 10), perhaps in part due to the higher overall prevalence of *MDM2* alterations in the EAS population (Supplementary Fig 1, Supplementary Data 1). Furthermore, in contrast to *KRAS*-altered tumors, *ATM* alterations showed mutual exclusivity with *EGFR* alterations in EUR, AFR, and EAS ancestries (Fig. 3c, Supplementary Data 11). Consistent with prior studies portraying the importance of β-catenin signaling in *EGFR*-altered NSCLC^30^, alterations in *CTNNB1*, the gene coding β-Catenin, which is the downstream effector of the Wnt signaling pathway, co-occurred with *EGFR* alterations across all ancestry groups, while being mutually exclusive with *KRAS* alterations (Figs. 2c, 3c, Supplementary Data 5, Supplementary Data 11).

Alterations in other genes such as *NFKBIA*, *CDK6*, *CDK4,* and *CCNE1* generally co-occurred with *EGFR* alterations across ancestry groups (Fig. 3c, Supplementary Data 11). Additionally, we also noted ancestry-specific patterns within *EGFR*-altered cases. For example, the EAS ancestry group showed high co-occurrence between *EGFR* and the Wnt pathway activating *APC* loss-of-function alterations (OR = 2.5, p = 2 × 10^−^^5^) (Fig. 3c, Supplementary Data 11). Amplifications of the hepatocyte growth factor (*HGF*) gene, which encodes a known ligand of the RTK receptor MET, were seen in 4–6% of *EGFR*-altered cases and were found to co-occur with *EGFR* alterations across all ancestry groups (Fig. 3b, c, Supplementary Data 10, Supplementary Data 11). In general, co-occurring alterations in cell cycle regulatory genes, *CDK4*, *CDK6*, *CCNE1,* and *CCND3* were common in *EGFR-*altered tumors across different ancestry groups (Fig. 3b, c, Supplementary Data 10, Supplementary Data 11). In contrast and as noted above, most cell cycle gene alterations were mutually exclusive with *KRAS* alterations across ancestries (Fig. 2c). *NKX2-1*, also known as thyroid transcription factor 1 (*TTF-1*), is a putative diagnostic marker in non-Sq NSCLC^31^. Interestingly, amplifications in *NKX2-1* showed significantly higher co-occurrence with *EGFR* as compared to *KRAS* alterations across all ancestries (Figs. 2c, 3c, Supplementary Data 5, Supplementary Data 11). As expected, amplifications of *NFKBIA*, present on the same amplicon as *NKX2-1*, also exhibited similar co-alteration and co-occurrence patterns (Figs. 2c, 3c, Supplementary Data 5, Supplementary Data 11). The observed *NKX2-1* co-alteration pattern is supportive of the proposed differential functional significance of *NKX2-1* in *EGFR* and *KRAS* mutated non-Sq NSCLC^32^.

In contrast to *KRAS*-altered non-Sq NSCLC, *EGFR*-altered tumors appeared to have a more conserved co-occurrence and mutual exclusivity alteration landscape across ancestry groups (Figs. 2c, 3c, Supplementary Data 5, Supplementary Data 11). Expectedly, known oncogenic activating alterations in *KRAS*, *BRAF*, *ALK,* and *ROS1* were mutually exclusive with *EGFR* alterations, similar to the observation in *KRAS*-altered non-Sq NSCLC (Fig. 3c, Supplementary Data 11).

Based on the differential overlap of *EGFR* short variants and amplifications across ancestry groups noted previously (Supplementary Fig. 2B), we also studied the differences in the co-occurrence and mutual exclusivity profile of the samples exclusively harboring *EGFR* short variants or *EGFR* amplifications. *EGFR* amplified samples showed strong co-occurrence of *TP53* alterations across all ancestry groups while being mutually exclusive in cases with *EGFR* short variants, particularly in the AFR group (Supplementary Fig. 5A, B, Supplementary Data 12, Supplementary Data 13). Similarly, *SMARCA4* alterations showed strong mutual exclusivity with EAS samples exclusively harboring *EGFR* short variants, while showing strong co-occurrence with EAS samples exclusively harboring *EGFR* amplifications (Supplementary Fig. 5A, B, Supplementary Data 12, Supplementary Data 13). Additionally, alterations in *NF1*, *MET*, *SMAD4*, *PTEN*, amplifications of 11q13 comprising *CCND1*, *FGF19*, *FGF3*, and *FGF4*, also showed differing co-occurrence and mutually exclusive patterns in samples with *EGFR* short variants and *EGFR* amplification in specific ancestry groups (Supplementary Fig. 5A, B, Supplementary Data 12, Supplementary Data 13).

Taken together, these data reveal a largely distinct co-alteration landscape between *KRAS* and *EGFR*-altered non-Sq NSCLC across ancestry groups and multiple overlapping as well as ancestry-specific co-occurring and mutually exclusive alterations within *KRAS-* and *EGFR*-altered non-Sq NSCLC.

### Immunotherapy-associated biomarkers differ based on ancestry in *KRAS* and *EGFR*-altered non-Sq NSCLC

**Fig. 4:**
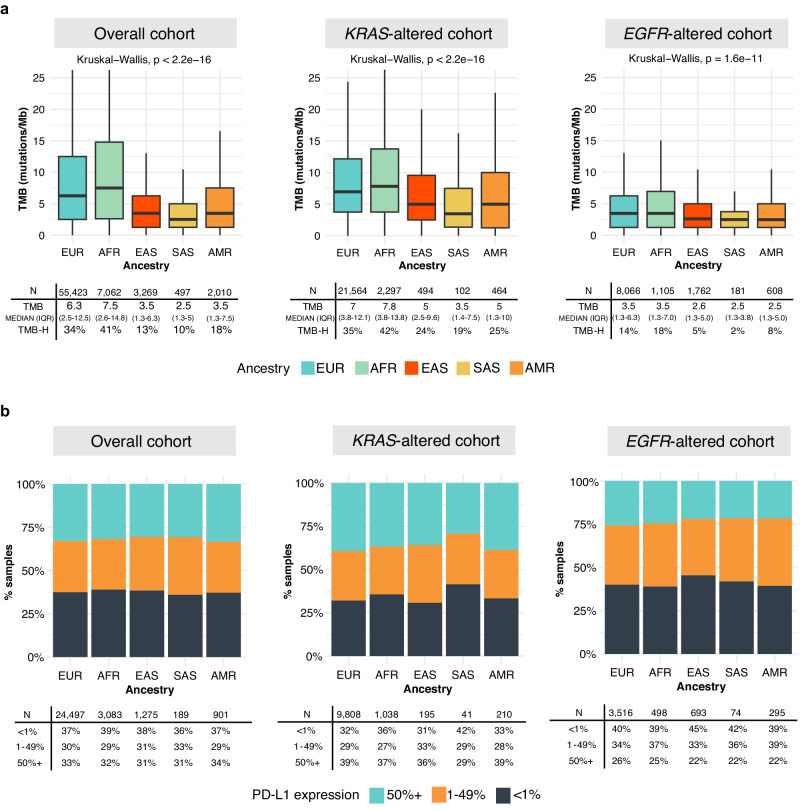
Patterns of immunotherapy-associated biomarkers, overall, and in *KRAS* and *EGFR*-altered non-Sq NSCLC based on ancestry. **a** Patterns of tumor mutational burden in the overall cohort, *KRAS*-altered and *EGFR*-altered non-Sq NSCLC based on ancestry. The color denotes the ancestry group. The total number of samples, the median TMB with the interquartile range (IQR), and the percentage of TMB-High (≥10 mutations/Mb) cases within each ancestry are also provided. Each box plot displays the interquartile range (IQR), with the lower and upper boundaries representing the 25th and 75th percentile; the line within the box represents the median and the whiskers extend to ±1.5 × IQR. **b** Patterns of PD-L1 positivity from DAKO 22C3 in the overall cohort, *KRAS*-altered and *EGFR*-altered non-Sq NSCLC based on ancestry. The color denotes the level of PD-L1 positivity: 50%+, 1–49%, and 0%. The total number of samples with available PD-L1 information and the percentage of PD-L1 positive cases within each ancestry are also provided. (EUR: European, AFR: African, EAS: East Asian, SAS: South Asian, AMR: Admixed American).

PD-L1/PD1 signaling suppresses T cell activation and PD-L1 expression has emerged as a predictive biomarker for immune checkpoint inhibitors (ICI). High tumor mutational burden (TMB), associated with elevated neoantigen load, has also been proposed as a biomarker for potential benefit from ICI, with the FDA approval of pembrolizumab in unresectable or metastatic solid tumors with a TMB ≥ 10 mutations/megabase (mut/Mb)^33^. Therefore, we explored the relationship of these immunotherapy-associated biomarkers among *KRAS-* and *EGFR-*altered cases based on ancestry. Across all non-Sq NSCLC, EUR, and AFR showed a significantly higher TMB and a higher proportion of TMB-high cases (≥10 mut/Mb) as compared to the EAS, SAS, and AMR ancestries (34–41% vs. 10–18%; Fig. 4a, *left*). Comparing *KRAS-* and *EGFR*-altered cohorts, we observed a lower TMB in *EGFR*-altered cases, consistent with *EGFR* being more common among non-smokers (Fig. 4a, *middle and right*). Interestingly, even within *KRAS-* and *EGFR*-altered cohorts, we continued to observe a higher TMB in EUR and AFR compared to the other ancestry groups (Fig. 4a, *middle and right*, Supplementary Data 14). The AFR ancestry showed the highest proportion of TMB-high cases in the overall cohort (41%) as well as in *KRAS-* (42%) and *EGFR*-altered (18%) non-Sq NSCLC. In contrast, SAS exhibited the lowest proportion of TMB-high cases in the overall cohort (10%) and in *KRAS-* (19%) and *EGFR*-altered (2%) non-Sq NSCLC (Fig. 4a, Supplementary Data 14).

Despite the difference in TMB, the levels of PD-L1 were largely similar across ancestry groups, where approximately 30% of the overall non-Sq NSCLC cohort had PD-L1 expression of ≥50%, with an additional 30% showing expression levels 1–49% (Fig. 4b). Compared to *KRAS*-altered tumors, *EGFR*-altered tumors showed a lower percentage of PD-L1 ≥ 50% but a higher percentage of PD-L1 expression in 1–49% range and in the PD-L1 negative group (<1%), with largely similar overall patterns across ancestry groups (Fig. 4b, *middle and right*, Supplementary Data 15).

Overall, the higher proportion of tumors with TMB-high status and PD-L1 ≥ 50% in *KRAS*-altered cases compared to *EGFR*-altered cases may impact the potential therapeutic strategies within these genetic subgroups.

## Discussion

Previous studies by our group and others have presented the landscape and the therapeutic relevance of allele-specific *KRAS* and *EGFR* alterations in non-sq NSCLC; yet, these studies have lacked or had limited information on ancestry-specific patterns^26,34,35^. Moreover, previous estimates of mutational prevalence, especially in minority patient populations, have been constrained by limited cohort size^36^. Through the examination of a large and diverse cohort, we provide unequivocal confirmation for previously published prevalence metrics, including the higher prevalence of *KRAS* alterations in EUR and AFR ancestries compared to EAS, SAS, and AMR ancestry groups and a higher prevalence of *EGFR* alterations in EAS, SAS and AMR compared to EUR and AFR ancestries^16,37,38^, while offering new insights on ancestry-specific patterns of gene alterations and immunotherapy-associated biomarkers.

Although smoking is generally associated with *KRAS*-altered but not *EGFR-*altered non-Sq NSCLC^39,40^, other factors, beyond environmental exposures^41^, may also contribute to the lower prevalence of *KRAS* alterations in EAS, SAS, and AMR ancestries. Supporting this, *EGFR* mutations are shown to be consistently more prevalent than *KRAS* mutations in the EAS ancestry regardless of smoking status^42^. Ancestral differences in the prevalence of *EGFR* and *KRAS* alterations are also driven by underlying ancestry-specific germline differences. This is supported by higher *EGFR* and lower *KRAS* mutation prevalence in Native American (NAT) and Hispanic/Latino populations, together comprising the AMR ancestry group, which includes components of the EAS ancestry, likely derived through waves of Asian-Pacific migration^43–46^. Moreover, independent of smoking status, NAT ancestry was shown to be associated with *EGFR* mutations^46,47^. The observed lower prevalence of *STK11* and *KEAP1* alterations in the *KRAS*-altered EAS and AMR as compared to the other ancestry groups may also stem from ancestry-specific germline variations, as both NAT and Hispanic/Latino NSCLC populations have been shown to exhibit a relatively lower prevalence of *STK11* and *KEAP1* alterations compared to EUR and Non-Hispanic White patients^45,46^. Interestingly, the SAS ancestry group appeared closer to EAS and AMR in the prevalence of *EGFR* and *KRAS* alterations but showed *STK11* and *KEAP1* co-alterations in *KRAS*-altered non-Sq NSCLC at a rate similar to those seen in EUR and AFR.

*STK11* and *KEAP1* alterations individually or together are poor prognostic factors in NSCLC and an enrichment of *STK11* and *KEAP1* alterations in *KRAS*-mutant non-Sq NSCLC has been previously reported^26,48^. Yet, the prevalence of these alterations in different ancestry groups had remained understudied^49,50^. The significantly lower co-alteration rate of *STK11* and *KEAP1* in *KRAS*-altered EAS and AMR as compared to other ancestries raises questions regarding potential clinical implications, including treatment outcomes with ICI and the recently emerging *KRAS* G12C inhibitors as discussed below. The differential co-mutation profile of *KRAS* G12C-mutant NSCLC tumors should be an important consideration when comparing clinical outcomes of different *KRAS* G12C inhibitors conducted in different regions of the world. It is noteworthy that the Asian patient population appears to derive a greater benefit from ICI as compared to non-Asian patients^51,52^. While different lifestyles, environmental exposures, and general physiological differences in different ancestry groups may contribute to variable treatment outcomes, a distinct co-alteration landscape in EAS compared to EUR and AFR ancestries may partly underlie a generally better prognosis in the former patient population. Moreover, although unlike PD-L1 levels, the predictive value of TMB for the use of ICI remains debated, the high mutational burden has been shown as a poor prognostic factor, and EAS non-Sq NSCLC have been shown to harbor more stable genomes as compared to EUR non-Sq NSCLC^42,53–55^. Together with a lower *STK11* and *KEAP1* co-alteration rate, higher genomic stability of the *KRAS*-mutant EAS ancestry group may explain better treatment outcomes compared to EUR and AFR ancestries. Conversely, no differential benefit with *EGFR* inhibitors has been observed in Asian patients with *EGFR*-mutant cancer compared to non-Asians^56^, which is consistent with a more conserved co-alteration landscape in *EGFR*-mutant non-Sq NSCLC across ancestries.

Targeted inhibition of mutant *EGFR* utilizing highly efficacious third-generation *EGFR* inhibitors has transformed patient care in NSCLC. Moreover, a subset of *KRAS*-altered non-Sq NSCLC, representing the biggest alteration subgroup in White and Black patient populations, has long been un-druggable but is now starting to benefit from mutant-specific *KRAS* G12C inhibitors^57^. However, unlike *EGFR*-mutant non-Sq NSCLC that derive impressive clinical benefit from *EGFR* inhibitors, thus far, *KRAS* G12C inhibitors have shown relatively less pronounced clinical activity in *KRAS* G12C-mutant non-Sq NSCLC^57–59^. A higher overall mutation burden and less conserved co-alteration landscape may partly explain this disparity in clinical activity, and it remains to be seen how distinct co-alteration patterns in *KRAS* G12C-mutant NSCLC across ancestries may influence the activity of *KRAS* G12C inhibitors. This underscores the importance of assessing the co-alteration landscape of these tumors and studying the potential impact of ancestry on clinical outcomes to *KRAS* G12C inhibitors.

Co-occurring *KEAP1*, *CDKN2A,* and *SMARCA4* loss-of-function alterations have recently emerged as poor prognostic biomarkers in *KRAS* G12C-mutant non-Sq NSCLC treated with the *KRAS* G12C inhibitors, sotorasib and adagrasib^60^. While *SMARCA4* alterations distributed rather evenly across the five ancestry groups we studied, a lower *KEAP1* co-alteration rate in EAS and AMR may associate with better treatment outcomes in these patient populations. Although *CDKN2A* alterations generally appear to be a poor prognostic factor, associated with worse outcomes not only with *KRAS* G12C inhibitors but also ICI and *EGFR* inhibitors^61,62^, the observed similar prevalence of *CDKN2A* co-alterations in both *KRAS* and *EGFR*-mutant non-Sq NSCLC across ancestry groups is unlikely to impact treatment outcomes across ancestry groups. These findings warrant further work to understand the impact of co-occurring alterations on the efficacy of different treatment modalities, especially in AFR, AMR, and SAS ancestries, which remain vastly understudied.

With a higher prevalence of actionable RTK alterations, EAS, SAS, and AMR might harbor a larger actionable population over EUR and AFR, deriving clinical benefit from multiple approved and effective RTK inhibitors. However, with a bigger proportion of PD-L1 ≥ 50% tumors, the *KRAS*-mutant EUR and AFR non-Sq NSCLC may derive additional benefit from ICI. Despite a relatively higher proportion of PD-L1 ≥ 50% tumors, higher *STK11* and *KEAP1* co-alterations in *KRAS*-mutant EUR and AFR are expected to attenuate the activity of ICI and therapy in general. Together, our findings unveil a complex web of molecular alterations that may impact the therapeutic susceptibility of *KRAS* and *EGFR-*mutant non-Sq NSCLC across different ancestry groups and consequently may contribute to molecular underpinnings of cancer disparities. A limitation of this study is the missing underlying clinical outcome data and treatment history, with the latter bearing possible implications for the observed genomic alteration landscape. Although our analysis included more than 300 cancer-relevant genes, additional parallels, and differences in the co-alteration landscape of *KRAS* and *EGFR*-mutant non-Sq NSCLC and across different ancestries are likely to continue to emerge along with future comprehensive sequencing efforts and evolving treatment landscape.

In summary, by informing the prevalence of *KRAS* and *EGFR* alterations, the associated genomic co-alterations, and patterns of immunotherapy-associated biomarkers among different ancestry groups, our findings offer the potential to help formulate new therapeutic hypotheses, propel evaluation of new therapeutic strategies, and influence public health policies that may aid alleviation of cancer disparities.

## Methods

### Comprehensive genomic profiling cohort of non-squamous NSCLC

The study cohort included 68,297 patients with non-squamous non-small cell lung cancer, who received comprehensive genomic profiling (CGP) using FoundationOne®/FoundationOne®CDx assays, as part of routine clinical care, through December 2022 from formalin-fixed, paraffin-embedded (FFPE) tumor biopsies. Approval for this study, including a waiver of informed consent and a Health Insurance Portability and Accountability Act waiver of authorization, was obtained from the WCG Institutional Review Board (Protocol No. 20152817). The Institutional Review Board granted a waiver of informed consent under 45 CFR § 46.116 based on review and determination that this research meets the following requirements: (i) the research involves no more than minimal risk to the subjects; (ii) the research could not practicably be carried out without the requested waiver; (iii) the waiver will not adversely affect the rights and welfare of the subjects. This study was performed in compliance with all relevant ethical regulations including the Declaration of Helsinki. No clinical or treatment information was available for the samples in this cohort.

Briefly, DNA was extracted from FFPE sections and CGP was performed on hybridization-captured, adapter ligation–based libraries for exons of ~324 (FoundationOne®CDx) and ~315 (FoundationOne®) cancer-related genes and select introns of genes frequently rearranged in cancer, as described previously^63,64^. Data analysis was limited to 296 genes commonly targeted on both assays. The reported genomic alterations included known or likely pathogenic short variants (base substitutions, small insertions/deletions), copy number alterations (gene amplifications of oncogenes, and homozygous deletions of tumor suppressors) as well as gene fusions and rearrangements predicted to activate oncogenes or inactivate tumor suppressors^63,64^, and included a multi-step detection procedure described in detail in our prior study^26^. Disease diagnosis included: lung adenocarcinoma (n = 53,661), lung non-small cell lung carcinoma (nos) (n = 12,533), lung large cell neuroendocrine carcinoma (n = 1246), lung sarcomatoid carcinoma (n = 527), lung large cell carcinoma (n = 276), lung carcinosarcoma (n = 54).

### Prediction of genetic ancestry

The genetic ancestry for each patient was predicted using a single nucleotide polymorphism (SNP)-based approach, from the targeted next-generation sequencing assay^15,65^^,^^67^^,68^. Briefly, >40,000 germline single nucleotide polymorphisms (SNPs) included in the targeted gene panel sequencing were overlapped with those captured in the phase 3 1000 Genomes Project and projected using principal component analysis to five principal components (PCs). These PCs were used to train a random forest classifier; the classifier performance was evaluated using ten-fold cross-validation performed on the 1000 genomes project cohort, as described previously^68^. Individuals were classified into the following ancestry groups based on the identified predominant ancestry: European, African, East Asian, South Asian, and admixed American. Comparison of genomic patterns in each ancestry group was performed using the European ancestry group as a reference.

### Detection of immunotherapy-related biomarkers

Tumor mutational burden, calculated as the number of non-driver synonymous and non-synonymous mutations across a ~0.8–1.2 megabase (Mb) region, using a prior validated approach^66^ was also reported as part of the CGP profiling. In addition, data on PD-L1 expression was available for 29,945 cases. PD-L1 expression was determined by immunohistochemistry (IHC) performed on FFPE tissue sections using the Dako 22C3 PD-L1 antibody, according to the manufacturer’s instructions (catalog number SK006). PD-L1 expression was binned into three categories based on the fraction of tumor cells staining with ≥1% intensity: negative (<1%), low positive (1–49%), or high positive (≥50%).

### Statistical methods and software

A comparison of the prevalence of gene alterations in the overall non-Sq NSCLC between the five ancestry groups was performed using a Chi-squared test. Similarly, to assess the difference in prevalence of each *KRAS* and *EGFR* alteration identified between the five ancestry groups, a Chi-squared test was used. P-values were calculated for each comparison and adjusted for multiple hypothesis correction using the Benjamini–Hochberg false discovery rate (FDR) procedure. A Chi-squared test was also used to compare the breakdown of the three different alteration categories (short variant only, amplification only, and short variant + amplification) in *KRAS-* and *EGFR-*altered non-Sq NSCLCs within each ancestry group to their breakdown in the EUR ancestry group. Differences in the prevalence of co-occurring gene alterations and immunotherapy-related biomarkers between ancestry groups in *KRAS*- and *EGFR*-altered NSCLCs were evaluated using a Fisher’s exact test. This was also specifically applied for comparing the prevalence of concomitant *KRAS, KEAP1*, and *STK11* alterations within each ancestry. Comparisons were made against the EUR group for each other ancestry subgroup. Two-sided P-values were calculated for each comparison and then adjusted for multiple hypothesis correction using the Benjamini–Hochberg FDR procedure. The distribution of TMB across the ancestry groups was compared using the Kruskal–Wallis test, for the overall cohort as well as for *KRAS-* and *EGFR-*altered non-Sq NSCLCs. For each ancestry subgroup, patterns of co-occurrence and mutual exclusivity between *KRAS* and other targeted genes in the panel, as well as between *EGFR* and other targeted genes in the panel were also evaluated using a Fisher’s exact test with FDR-based correction. Statistical significance was set at an FDR-corrected p ≤ 0.05.

### Supplementary information

Supplementary Data 1: Prevalence of gene alterations in non-Sq NSCLC based on ancestry.

Supplementary Data 2: Prevalence of different *KRAS* alterations based on ancestry.

Supplementary Data 3: Prevalence of different *EGFR* alterations based on ancestry.

Supplementary Data 4: Prevalence of co-occurring gene alterations in *KRAS*-altered non-Sq NSCLC based on ancestry.

Supplementary Data 5: Patterns of co-occurrence and mutual exclusivity between *KRAS* and other gene alterations in different ancestry groups.

Supplementary Data 6: Prevalence of co-occurring alterations in *KRAS*, *KEAP1*, and *STK11* in different ancestry groups.

Supplementary Data 7: Breakdown of co-occurring *EGFR* and *KRAS* alterations in samples of East Asian ancestry

Supplementary Data 8: Patterns of co-occurrence and mutual exclusivity between samples with *KRAS* short variants only and other gene alterations in the overall cohort and in different ancestry groups.

Supplementary Data 9: Patterns of co-occurrence and mutual exclusivity between samples with *KRAS* amplifications only and other gene alterations in the overall cohort and in different ancestry groups.

Supplementary Data 10: Prevalence of co-occurring gene alterations in *EGFR*-altered non-Sq NSCLC based on ancestry.

Supplementary Data 11: Patterns of co-occurrence and mutual exclusivity between *EGFR* and other gene alterations in different ancestry groups.

Supplementary Data 12: Patterns of co-occurrence and mutual exclusivity between samples with *EGFR* short variants only and other gene alterations in the overall cohort and in different ancestry groups.

Supplementary Data 13: Patterns of co-occurrence and mutual exclusivity between samples with *EGFR* amplifications only and other gene alterations in the overall cohort and in different ancestry groups.

Supplementary Data 14: Prevalence of TMB-high cases among *KRAS* and *EGFR*-altered cases.

Supplementary Data 15: Prevalence of PD-L1 expression status among *KRAS* and *EGFR*-altered cases.

Supplementary Information

## Data Availability

The sequencing data utilized for this study are derived from clinical samples. All the relevant data supporting the findings of this study are provided within the main manuscript and its supplementary files. Due to HIPAA requirements, we are not authorized to share individualized patient genomic data, which contains potentially identifying or sensitive patient information. Foundation Medicine is committed to collaborative data analysis, with well-established, and widely utilized mechanisms by which investigators can query our core genomic database of >700,000 de-identified sequenced cancers to obtain aggregated datasets. For more information and mechanisms of access, please contact the corresponding author(s) or the Foundation Medicine, Inc. Data Governance Council at data.governance.council@foundationmedicine.com.

## Code availability

Statistics and computation were carried out using *chi2_contingency* and *fisher_exact* functions within the SciPy package (version 1.2.1) in Python 2.7.18 (Python Software Foundation). Plotting was carried out using the ggpubr (version 0.6.0) and ggplot2 (version 3.4.4) packages in R 4.3.1 (R Foundation for Statistical Computing). Code for generating figures is available upon request.
