# Supplementary material for: Ancestry-associated co-alteration landscape of KRAS and EGFR-altered non-squamous NSCLC
Source: NPJ Precis Oncol. 2024 Jul 20;8:153. doi: 10.1038/s41698-024-00644-4 (PMC11271287; doi:10.1038/s41698-024-00644-4)
Supplement: Supplementary file 16 — Supplementary Information [file 41698_2024_644_MOESM16_ESM.pdf]

Supplementary Figure 1. Prevalence of gene alterations in the overall non-Sq NSCLC cohort based on ancestry.

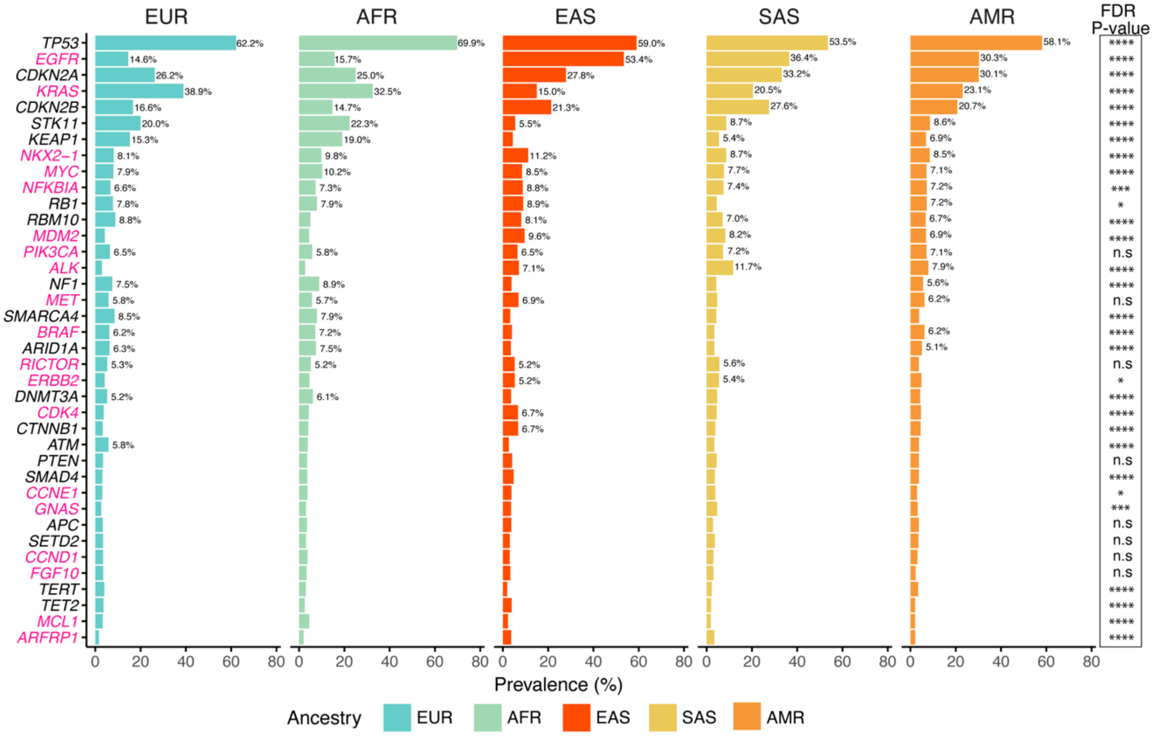

Barplot showing the prevalence of the top gene alterations across different ancestry subgroups in the overall non-Sq NSCLC cohort based on ancestry. A union of the 30 most common gene alterations in each ancestry group, accounting for a total of 38 genes is plotted. The color of each bar denotes the ancestry group. Genes predicted to be oncogenes are shown in pink. (EUR: European, AFR: African, EAS: East Asian, SAS: South Asian, AMR: Admixed American). Statistically significant patterns, determined by a Chi-squared test followed by FDR correction, are shown with the following *P*-value thresholds: * 0.05, ** 0.01, *** 0.001, **** 0.0001, n.s, not significant.

Supplementary Figure 2. Overlap of short variants and amplifications within *KRAS* and *EGFR* in non-Sq NSCLC.

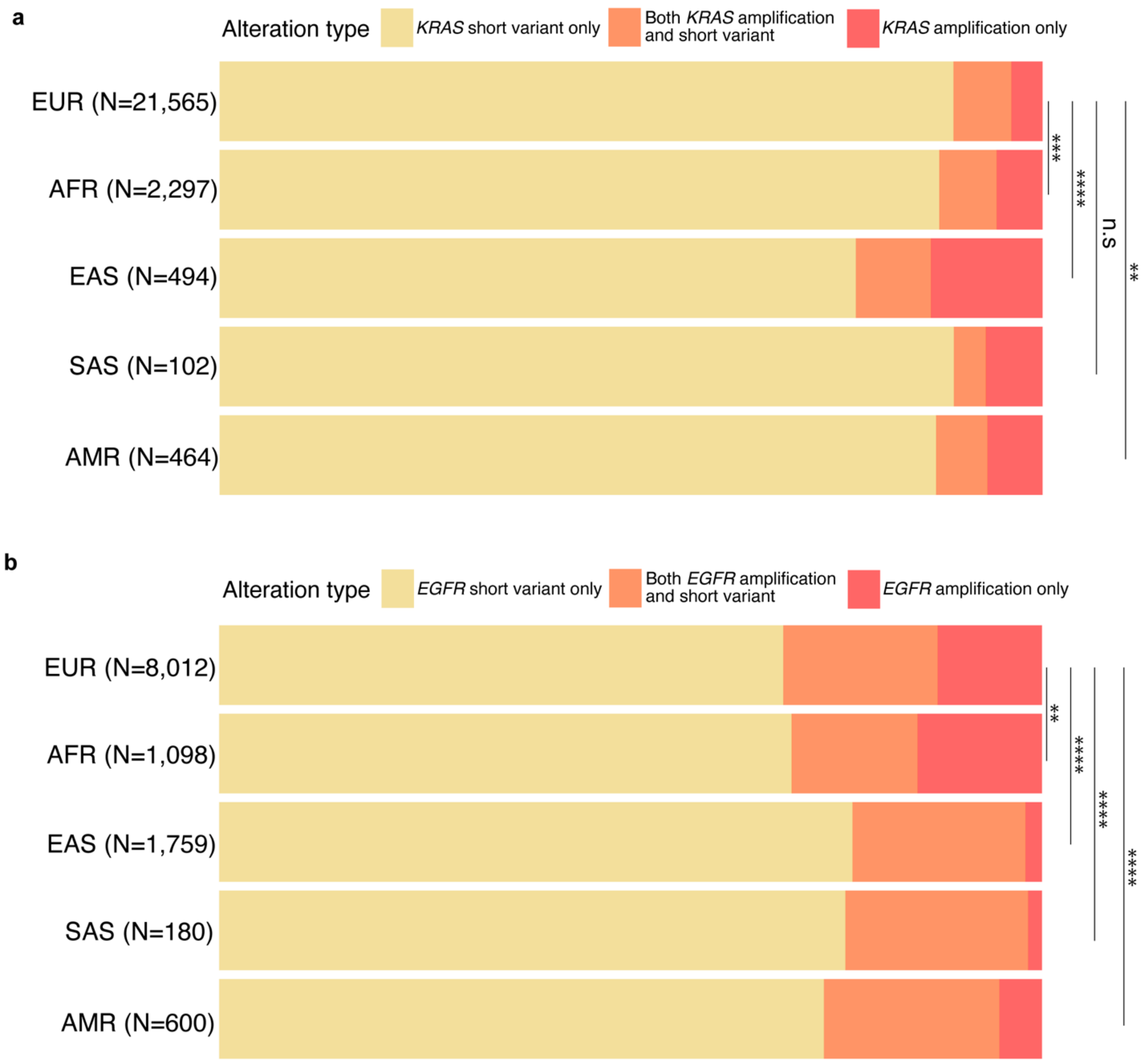

a. Breakdown of cases with *KRAS* short variants only, amplifications only, and cases with both classes of alterations within each ancestry group. The total number of *KRAS*-altered cases within each ancestry group is provided. Statistically significant patterns, determined by a Chi-

squared test, are shown with the following *P*-value thresholds: * 0.05, ** 0.01, *** 0.001, **** 0.0001, n.s, not significant.

b. Breakdown of cases with *EGFR* short variants only, amplifications only, and cases with both classes of alterations within each ancestry group. The total number of *EGFR*-altered cases within each ancestry group is provided. Statistically significant patterns, determined by a Chi-squared test, are shown with the following *P*-value thresholds: * 0.05, ** 0.01, *** 0.001, **** 0.0001, n.s, not significant.

Supplementary Figure 3. Prevalence of concomitant *KRAS, KEAP1* and *STK11* alterations across ancestry groups.

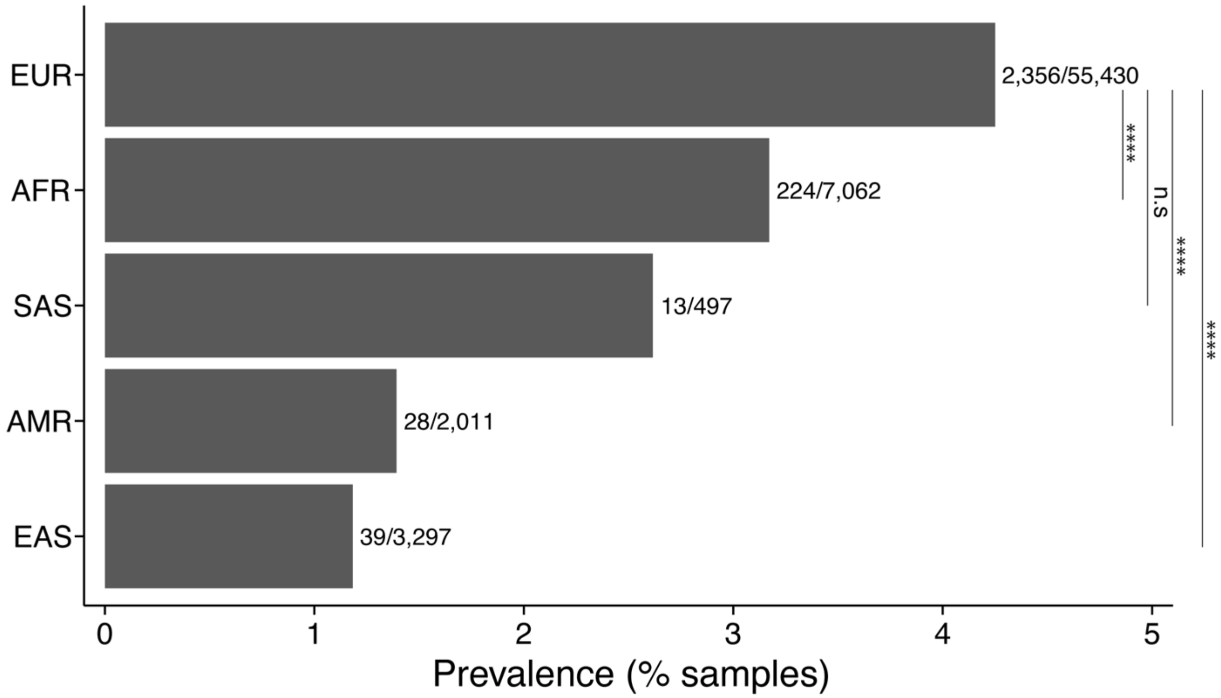

Barplot displaying the prevalence of cases, as a percentage of total cases (N), with co-occurring alterations in *KRAS*, *KEAP1,* and *STK11* in each ancestry group. (EUR: European, AFR: African, EAS: East Asian, SAS: South Asian, AMR: Admixed American). Statistically significant patterns, determined by a Fisher's exact test, are shown with the following *P*-value thresholds: * 0.05, ** 0.01, *** 0.001, **** 0.0001, n.s, not significant.

Supplementary Figure 4. Co-occurrence and mutual exclusivity of gene alterations in cases with *KRAS* short variants and *KRAS* amplifications exclusively, across ancestry groups.

a

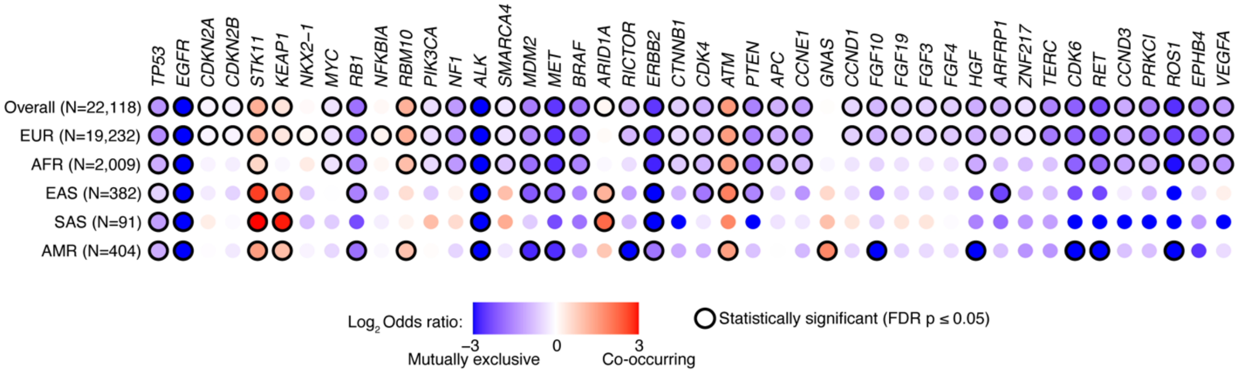

b

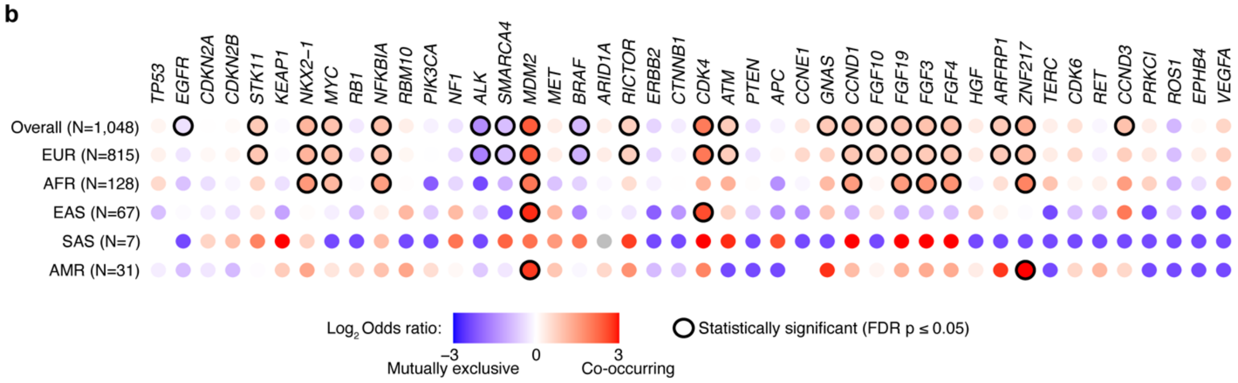

a. Patterns of co-occurrence and mutual exclusivity between *KRAS* short variants and other gene alterations in each ancestry. Statistically significant patterns, determined by a Fisher's exact test with FDR correction (FDR $p \leq 0.05$), are highlighted with a black circle. Patterns of co-occurrence are shown in shades of red while patterns of mutual exclusivity are shown in shades of blue.

b. Patterns of co-occurrence and mutual exclusivity between *KRAS* amplifications and other gene alterations in each ancestry. The same set of genes shown in panel A are shown here. Statistically significant patterns, determined by a Fisher's exact test with FDR correction (FDR

$p \leq 0.05$), are highlighted with a black circle. Patterns of co-occurrence are shown in shades of red while patterns of mutual exclusivity are shown in shades of blue.

Cases with co-occurring *KRAS* amplifications and short variants were excluded from these analyses. (EUR: European, AFR: African, EAS: East Asian, SAS: South Asian, AMR: Admixed American). Gene alterations showing statistically significant co-occurrence/mutual exclusivity with either *KRAS* short variants only or *KRAS* amplifications only, in at least one ancestry and with evaluable data across all ancestry groups are plotted.

Supplementary Figure 5. Co-occurrence and mutual exclusivity of gene alterations in cases with *EGFR* short variants and *EGFR* amplifications exclusively, across ancestry groups.

a

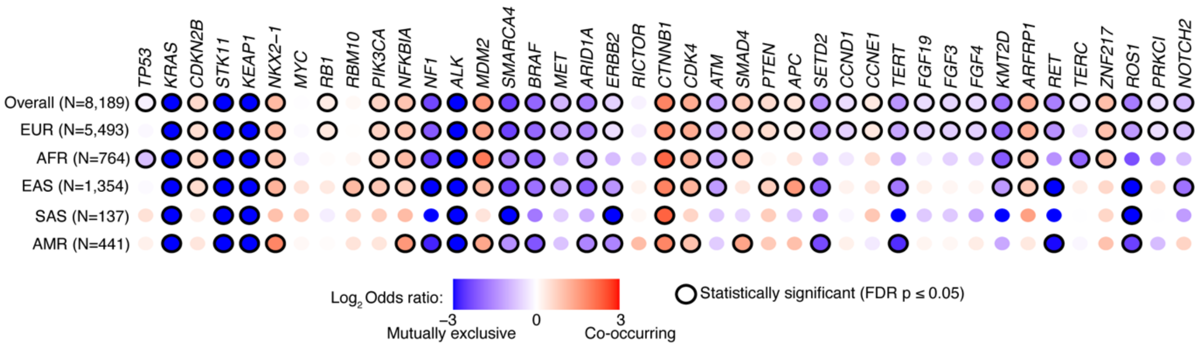

b

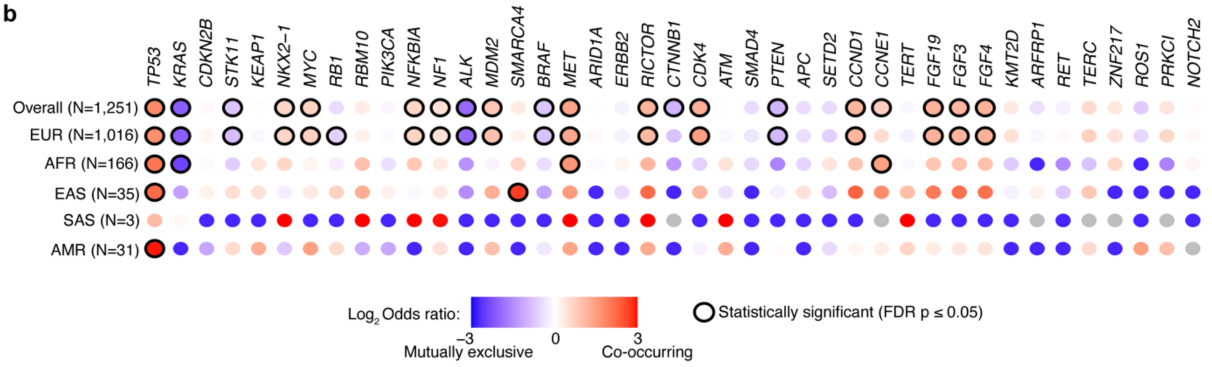

a. Patterns of co-occurrence and mutual exclusivity between *EGFR* short variants and other gene alterations in each ancestry. Statistically significant patterns, determined by a Fisher's exact test with FDR correction (FDR $p \leq 0.05$), are highlighted with a black circle. Patterns of co-occurrence are shown in shades of red while patterns of mutual exclusivity are shown in shades of blue.

b. Patterns of co-occurrence and mutual exclusivity between *EGFR* amplifications and other gene alterations in each ancestry. Statistically significant patterns, determined by a Fisher's exact test with FDR correction (FDR $p \leq 0.05$), are highlighted with a black circle. Patterns of

co-occurrence are shown in shades of red while patterns of mutual exclusivity are shown in shades of blue.

Cases with co-occurring *EGFR* amplifications and short variants were excluded from these analyses. (EUR: European, AFR: African, EAS: East Asian, SAS: South Asian, AMR: Admixed American). Gene alterations showing statistically significant co-occurrence/mutual exclusivity with either *KRAS* short variants only or *KRAS* amplifications only, in at least one ancestry and with evaluable data across all ancestry groups are plotted.

Supplementary Data

Supplementary Data 1: Prevalence of gene alterations in non-Sq NSCLC based on ancestry.

EUR: European, AFR: African, EAS: East Asian, SAS: South Asian, AMR: Admixed American

Supplementary Data 2: Prevalence of different *KRAS* alterations based on ancestry.

EUR: European, AFR: African, EAS: East Asian, SAS: South Asian, AMR: Admixed American

Supplementary Data 3: Prevalence of different *EGFR* alterations based on ancestry.

EUR: European, AFR: African, EAS: East Asian, SAS: South Asian, AMR: Admixed American

EGFR_ex_19_del: EGFR exon 19 deletions

EGFR_ex_19_ins: EGFR exon 19 insertions

EGFR_ex_20_ins: EGFR exon 20 insertions

Supplementary Data 4: Prevalence of co-occurring gene alterations in *KRAS*-altered non-Sq NSCLC based on ancestry.

EUR: European, AFR: African, EAS: East Asian, SAS: South Asian, AMR: Admixed American

For each gene, the prevalence within each ancestry was compared against EUR.

Supplementary Data 5: Patterns of co-occurrence and mutual exclusivity between *KRAS* and other gene alterations in different ancestry groups.

EUR: European, AFR: African, EAS: East Asian, SAS: South Asian, AMR: Admixed American

In each ancestry group, co-occurrence and mutual exclusivity was limited to genes altered in at least 10 cases within that ancestry cohort.

Supplementary Data 6: Prevalence of co-occurring alterations in *KRAS, KEAP1* and *STK11* in different ancestry groups.

EUR: European, AFR: African, EAS: East Asian, SAS: South Asian, AMR: Admixed American

Prevalence within each ancestry was compared against EUR.

Supplementary Data 7: Breakdown of co-occurring *EGFR* and *KRAS* alterations in samples of East Asian ancestry

Supplementary Data 8: Patterns of co-occurrence and mutual exclusivity between samples with *KRAS* short variants only and other gene alterations in the overall cohort and in different ancestry groups.

KRAS short variants include base substitutions and short insertions and deletions. This analysis includes samples with only *KRAS* short variants; samples with concurrent *KRAS* amplifications were excluded.

EUR: European, AFR: African, EAS: East Asian, SAS: South Asian, AMR: Admixed American

Within each ancestry group, co-occurrence and mutual exclusivity was limited to genes altered in at least 10 cases in the overall cohort.

Supplementary Data 9: Patterns of co-occurrence and mutual exclusivity between samples with *KRAS* amplifications only and other gene alterations in the overall cohort and in different ancestry groups.

This analysis includes samples with only *KRAS* amplifications; samples with concurrent *KRAS* short variants were excluded.

EUR: European, AFR: African, EAS: East Asian, SAS: South Asian, AMR: Admixed American

In each ancestry group, co-occurrence and mutual exclusivity was limited to genes altered in at least 10 cases within that ancestry cohort.

Supplementary Data 10: Prevalence of co-occurring gene alterations in *EGFR*-altered non-Sq NSCLC based on ancestry.

EUR: European, AFR: African, EAS: East Asian, SAS: South Asian, AMR: Admixed American

For each gene, the prevalence within each ancestry was compared against EUR.

Supplementary Data 11: Patterns of co-occurrence and mutual exclusivity between *EGFR* and other gene alterations in different ancestry groups.

EUR: European, AFR: African, EAS: East Asian, SAS: South Asian, AMR: Admixed American

In each ancestry group, co-occurrence and mutual exclusivity was limited to genes altered in at least 10 cases within that ancestry cohort.

Supplementary Data 12: Patterns of co-occurrence and mutual exclusivity between samples with *EGFR* short variants only and other gene alterations in the overall cohort and in different ancestry groups.

EGFR short variants include base substitutions and short insertions and deletions. Samples with concurrent *EGFR* amplification were excluded for this analysis.

EUR: European, AFR: African, EAS: East Asian, SAS: South Asian, AMR: Admixed American

In each ancestry group, co-occurrence and mutual exclusivity was limited to genes altered in at least 10 cases within that ancestry cohort.

Supplementary Data 13: Patterns of co-occurrence and mutual exclusivity between samples with *EGFR* amplifications only and other gene alterations in the overall cohort and in different ancestry groups.

This analysis includes samples with only *EGFR* amplifications; samples with concurrent *EGFR* short variants were excluded.

EUR: European, AFR: African, EAS: East Asian, SAS: South Asian, AMR: Admixed American

In each ancestry group, co-occurrence and mutual exclusivity was limited to genes altered in at least 10 cases within that ancestry cohort.

Supplementary Data 14: Prevalence of TMB-high cases among *KRAS* and *EGFR* altered cases.

EUR: European, AFR: African, EAS: East Asian, SAS: South Asian, AMR: Admixed American

Tumor mutational burden (TMB); TMB High is defined at ≥ 10 mutations/Megabase and TMB Low is defined at < 10 mutations/Megabase

Prevalence in each ancestry was compared against EUR.

Supplementary Data 15: Prevalence of PD-L1 expression status among *KRAS* and *EGFR* altered cases.

EUR: European, AFR: African, EAS: East Asian, SAS: South Asian, AMR: Admixed American

PD-L1 expression was binned into three categories based on fraction of tumor cells staining with >=1% intensity. <1%; 1-49% and 50%+

Each ancestry was compared against EUR.
